# Development and optimization of a hybridization technique to type the classical class I and class II B genes of the chicken MHC

**DOI:** 10.1007/s00251-019-01149-2

**Published:** 2019-11-25

**Authors:** Nicola D. Potts, Coraline Bichet, Laurence Merat, Edouard Guitton, Andrew P. Krupa, Terry A. Burke, Lorna J. Kennedy, Gabriele Sorci, Jim Kaufman

**Affiliations:** 1 Department of Pathology, University of Cambridge, Tennis Court Road, Cambridge, CB2 1QP UK; 2 LGC Ltd., Newmarket Road, Fordham, Ely, CB7 5WW UK; 3 BioGéoSciences, CNRS UMR 5561, Université de Bourgogne Franche-Comté, 6 Boulevard Gabriel, 21000 Dijon, France; 4 Institute of Avian Research, An der Vogelwarte 21, 26386 Wilhelmshaven, Germany; 5 Plate-Forme d’Infectiologie Expérimentale (PFIE), UE-1277, INRA Centre Val de Loire, 37380 Nouzilly, France; 6 Department of Animal and Plant Sciences, University of Sheffield, Western Bank, S10 2TN Sheffield, UK; 7 Division of Population Health, Health Services Research & Primary Care, University of Manchester, Oxford Road, M13 9PL Manchester, UK; 8 Department of Veterinary Medicine, University of Cambridge, Madingley Road, Cambridge, CB3 0ES UK

**Keywords:** Avian, BLB1, BLB2, BF1, BF2, Recombination

## Abstract

The classical class I and class II molecules of the major histocompatibility complex (MHC) play crucial roles in immune responses to infectious pathogens and vaccines as well as being important for autoimmunity, allergy, cancer and reproduction. These classical MHC genes are the most polymorphic known, with roughly 10,000 alleles in humans. In chickens, the MHC (also known as the BF-BL region) determines decisive resistance and susceptibility to infectious pathogens, but relatively few MHC alleles and haplotypes have been described in any detail. We describe a typing protocol for classical chicken class I (BF) and class II B (BLB) genes based on a hybridization method called reference strand-mediated conformational analysis (RSCA). We optimize the various steps, validate the analysis using well-characterized chicken MHC haplotypes, apply the system to type some experimental lines and discover a new chicken class I allele. This work establishes a basis for typing the MHC genes of chickens worldwide and provides an opportunity to correlate with microsatellite and with single nucleotide polymorphism (SNP) typing for approaches involving imputation.

## Introduction

The major histocompatibility complex (MHC) was defined in humans and mice as the genetic locus responsible for the fastest rejection of tissue allografts (Klein 1986), but decades of work has shown that these regions are large and complex, determining many different functions of biological and medical importance (Trowsdale and Knight 2014; Scepanovic et al. 2018). Foremost of these functions are those determined by the so-called classical class I and class II molecules, which are encoded by the most polymorphic loci in the human genome. The genes for such molecules are found throughout the jawed vertebrates, and are typically highly polymorphic (Kaufman 2018a).

In mammals, these classical MHC molecules play crucial roles in both adaptive and innate immune responses. Classical class I molecules are expressed on most cells and bear peptides derived from proteins in the cytoplasm and nucleus (where viruses and a few intracellular bacteria replicate); such class I molecules are typically recognized by CD8-bearing cytotoxic T lymphocytes (CTL) that kill the pathogen-infected cells (Van Kaer 2002). In addition, some of the classical class I molecules are recognized by receptors on natural killer (NK) cells that deliver inhibitory signals, with the NK cell killing the target cell if the inhibitory signal is not received (Bryceson et al. 2011). In contrast, classical class II molecules are expressed by professional antigen presenting cells (including macrophages, dendritic cells and B lymphocytes) as well as being induced by inflammatory signals on a variety of cell types, and bear peptides derived from proteins in intracellular vesicles and from the extracellular space where bacteria and parasites are common. Such classical class II molecules are typically recognized by several subsets of CD4-bearing T cells (so-called Th1, Th2, Th17, Treg, among others) with a variety of nuanced responses appropriate to the particular pathogen (Kaplan et al. 2015). These classical MHC molecules are also important in responses to tumors, as well as being the principle genes involved in autoimmunity and allergy (Trowsdale and Knight 2014; Scepanovic et al. 2018).

The most striking feature of the classical MHC molecules is their enormous polymorphism, with over 10,000 alleles described in humans (Robinson et al. 2015). The polymorphic amino acid positions in these molecules are largely located in the peptide-binding groove, so that different MHC alleles bind different repertoires of peptides. In class I molecules, a peptide-binding groove is formed by the α1 and α2 domains of the heavy (or α) chain (encoded by exons 2 and 3 of the class I genes). In class II molecules, such a peptide-binding groove is formed by the α1 domain of the heavy (or α) chain together with the β1 domain of the light (β) chain of class II molecules (encoded by exon 2 of both genes) (Yaneva et al. 2010). This high polymorphism of MHC molecules is thought to be driven by a molecular arms race between host and pathogens (Spurgin and Richardson 2010; Phillips et al. 2018) in which the pathogen mutates amino acids so that peptides are no longer bound by particular MHC molecules, which is counteracted by the emergence of new MHC molecules, and so on. Recognition of class I molecules by NK cells is also dependent of portions of the bound peptide (Cassidy et al. 2014, 2015), so the peptide-binding specificity is of crucial importance.

Chickens (also called domestic fowl) are beset by many economically-important infectious diseases, and the B blood group was found to determine enormous resistance and susceptibility (Miller and Taylor 2016). Now it is known that the B locus determines this blood group and includes the BF-BL region that is the MHC (as defined as the locus that confers rapid tissue allograft resistance), which is particularly compact and simple (Kaufman et al. 1995, 1999; Kaufman 2018b). There are many experimental challenges and unbiased field studies showing that resistance and susceptibility to certain viruses, bacteria and even parasites can be determined by the B locus (Boonyanuwat et al. 2006; reviewed in Kaufman et al. 1995; Kaufman 2018b; Miller and Taylor 2016). At least part of these strong genetic associations with resistance and susceptibility is due to the fact that the chicken MHC expresses only one class I molecule (with the heavy chain from the BF2 locus) and one class II molecule (with the light chain from the BLB2 locus) at a high level and throughout the body (Kaufman et al. 1995, 1999; Kaufman 2018b; Wallny et al. 2006; Parker and Kaufman 2017). However, there is another polymorphic class I molecule (with the heavy chain from the BF1 locus) that is only poorly expressed and thought to be an NK ligand (much like HLA-C) (Ewald and Livant 2004; Kim et al. 2018) as well as a second polymorphic class II molecule (with the light chain from the BLB1 locus) that is only well-expressed in the intestine (Parker and Kaufman 2017). In addition, there are many other polymorphic genes within the chicken MHC (Atkinson et al. 2001; Chattaway et al. 2016; Chazara et al. 2011; Goto et al. 2009; van Hateren et al. 2013; Hosomichi et al. 2008; Rogers and Kaufman 2008; Walker et al. 2005, 2011) as well as in the TRIM and BG regions next to it that may contribute to disease resistance ascribed to the B locus.

There have been enormous efforts over decades to type human MHC genes in order to correlate alleles with transplantation and disease phenotypes. Currently, most genes in the human genome are relatively easily and cheaply typed by single nucleotide polymorphisms (SNPs) (Hirschhorn and Gajdos 2011), but the density of allelic differences precludes binding a conserved oligonucleotide to the DNA encoding the peptide-binding regions so that the usual methods for typing SNPs are not possible for MHC genes. Instead, human MHC alleles are usually imputed by constellations of flanking SNPs (Dilthey et al. 2011, Moutsianas and Gutierrez-Achury 2018), but the sequences of such alleles need to be known and their flanking regions characterized in order to type by SNP imputation.

Such typing of chicken MHC genes would facilitate understanding of and selective breeding for resistance to economically important infectious diseases among chickens throughout the world, estimated by some authorities as many as 80 billion in any 1 year. Current methods include microsatellite markers and an MHC-focused SNP panel, both of which have been used extensively to type the MHC (Iglesias et al. 2019; Fulton et al. 2006, 2016a, 2016b; McElroy et al. 2005; Nguyen-Phuc et al. 2016). The advantages of these approaches are clear: they are relatively simple, high through-put and definitive. However, there are several disadvantages, chief among which is the fact that both sets of markers are anonymous and generally have not been related to the surrounding MHC genes (for which considerable biochemical and biological insight is available). In fact, the only well-characterized alleles have been from several experimental lines derived from egg-layer or general-purpose chickens (Hosomichi et al. 2008; Kaufman et al. 1999; reviewed in Miller et al. 2004). Thus far, these well-defined alleles of BLB1, BLB2, BF1 and BLF2 are found in relatively stable haplotypes, with names like B2 and B4. There are some (mostly partial sequences) of alleles from other lines and populations in the literature (Ewald and Livant 2004; Lima-Rosa et al. 2004; Livant and Ewald 2004; Livant et al. 2004), but these have never been examined in greater detail or added to the list of known haplotypes.

Therefore, we began a typing effort (Worley et al. 2008; Potts 2016) using a hybridization technique called reference strand-mediated conformation analysis (RSCA), originally developed for large-scale typing of human MHC genes (Argüello et al. 1998). Since that beginning, we have typed over 12,000 chickens from a wide variety of commercial lines, fancy breeds and indigenous chickens, but none of this work is yet published and even the original typing efforts are not available in a readily accessible publication. In this report, we adapt, optimize, validate with known experimental lines and finally analyze new experimental lines using the RSCA typing of chicken BLB1, BLB2, BF1 and BF2 genes, laying the foundation for reporting further results in the future.

## Materials and methods

### Samples

Genomic DNA from MHC-homozygous chicken lines 6_1,_ 7_2_ (both MHC haplotype B2), C-B4 (B4), C-B12 (B12), 15l (B15), P2a (B19), 0 and N (both B21) (bred and maintained then at the Institute for Animal Health, Compton, UK, but now available at the Roslin Institute, Easter Bush, U. K.) has been described (Shaw et al. 2007). Genomic DNA was provided from the experimental white leghorn lines LD, B21, B19, B13 and PA12, bred and maintained at the animal facilities at the Plate-Forme d’Infectiologie Expérimentale (PFIE), UE-1277, INRA Centre Val de Loire, Nouzilly, France (10.15454/1.5572352821559333e12). Samples were stored at 4 °C in 10 mM TrisCl, 1 mM ethylene diamine tetraacetic acid (EDTA), pH 7.5 (TE). Cloned genomic DNA of chicken class I and class II B genes used for production of fluorescently-labelled reference strands (FLRs) have been described (Jacob et al. 2000; Shaw et al. 2007). Nucleic acid concentrations were determined using a Nanodrop spectrophotometer (Thermo Scientific) with the dsDNA program.

### Polymerase chain reaction (PCR)

Amplifications were performed in a Biorad Tetrad 2 Peltier Thermal Cycler using an initial denaturing step of 1–2 min at 96 °C, then 30 cycles of 1 min denaturation at 96 °C, 1 min annealing at 59 °C (BLB genomic), 60 °C (FLR) or 63 °C (BF genomic), 1 min extension at 72 °C and at the end a final extension at 72 °C for 10 min. The amplifications were performed in 25 μl reactions with 1× reaction buffer (containing 2.5 mM MgCl_2_), 100 mM dNTPs (25 mM each of dATP, dCTP, dGTP and dDTP), primers, DNA and 0.5 units of Velocity DNA polymerase (Bioline). The primers (Worley et al. 2008) for class II B exon 2 were OL284BL (GTGCCCGCAGCGTTCTTC, also called UC54) and RV280BL (TCCTCTGCACCGTGAAGG, also called UC55) and for class I exon 2 to exon 3 were C71 (CGAGCTCCATACCCTGCGGTAC) and C75 (CTCCTGCCCAGCTCAGCCTTC) (MWG Biotech or Sigma), resuspended in TE at 100 pmol/μl and stored at − 20 °C and in the dark for 5(6)-carboxyfluorescein (FAM)-labelled primers. To make FLRs, plasmid DNA at 5 ng/μl TE was amplified with 400 nM forward primer and 4 μM reverse FAM-labelled primer. For experimental samples, genomic DNA at 15 ng/μl TE was amplified with 400 nM each primer and 4% DMSO.

### Purification of PCR products

The products from some PCR reactions were separated by gel electrophoresis using 1% UltraPure Agarose (Invitrogen) in 40 mM Tris, 20 mM acetic acid, 1 mM EDTA (1× TAE, pH 8) buffer with GelRed dye (Biotium) at 1/40,000, visualized using a FluroChem fluorescent imager (Alpha Innotech) and excised, followed by purification using the QIAquick Gel Extraction Kit (Qiagen). The products of other PCR reactions were purified directly using QIAquick PCR-Purification Kit (Qiagen). Briefly, the gel slices or reaction mixtures were dissolved in high-salt buffer supplied with the kits and applied to a silica spin column, which was washed with buffers supplied with the kits, and pure DNA was subsequently eluted in nuclease-free water.

### RSCA

Purified PCR products of each FLR were diluted in nuclease-free water to 5 ng/μl and mixed with an equal volume of the respective gene PCR product from genomic samples. The hybridization mix was denatured at 95 °C for 15 min in a Biorad Tetrad 2 Peltier Thermal Cycler, cooled by ramping down at 1 °C/s until 55 °C, held for 15 min, reduced to 4 °C for 15 min to stabilize the duplexes, diluted with an equal volume of nuclease-free water and stored on ice. Class I and class II B hybridization mixes were combined at a ratio of 4:1 BF/BLB in a fresh plate with 0.2 μl GeneScan 2500-ROX Size Standard (Applied Biosystems) and 9.8 μl nuclease-free water, for a total volume of 15 μl.

Plates were then run on an ABI PRISM® 3100 Genetic Analyzer (Applied Biosystems). DNA duplexes were separated using 50 cm capillaries with 4% non-denaturing CAP mixture, using an 8 kV injection voltage and 30 s injection time and a 1 kV run voltage, 30 °C run temperature and a 4000 s run time. CAP mixture was stored at 4 °C and prepared fresh every 2 weeks [12.5 g of 9% Conformational Analysis Polymer (Applied Biosystems), 5.4 g urea (Merck), 1.7 g sucrose (Merck), 1.125 g of 20× TTE (Tris-Taurine-EDTA buffer, National Diagnostic) and 1.8 g deionized water].

Data was imported into GeneMapper Software v3.7 (Applied Biosystems) for analysis, with migration values of allele peaks scored by the software relative to the internal size standard. Peak scores were sorted and grouped according to shared scores.

### Cloning, sequencing and analysis

Using the CloneJet kit (Fermentas), purified PCR products were treated to remove overhanging 3’ and 5’ nucleotides and incubated with T4 DNA ligase in the presence of cut pJET1.2/blunt-end vector. The DNA in the ligation mix was transformed into DH5α *E. coli* (made chemically-competent using the Hanahan method) by the heat shock method (Maniatis et al. 1982); selection was by disruption of a lethality gene in pJET1.2 and by growth overnight at 37 °C on lysogeny broth (LB) agar plates containing 100 μg/ml ampicillin*.*

Colonies were screened by PCR for the correct size insert using BioMix Red polymerase ready-mix (Bioline) in 15 μl reactions with 10 μM pJET1.2 forward and reverse primers as described in the CloneJet kit (Fermentas). Amplification was performed with an initial denaturation step at 95 °C for 3 min, followed by 25 cycles of 94 °C for 30 s, 60 °C for 30 s and 72 °C for 1 min/kb and finished by 72 °C extension for 3 min. Products were run on an agarose gel and corresponding positive colonies grown overnight in LB media containing 100 μg/ml ampicillin at 37 °C with shaking. Using the GenCatch Plasmid DNA Miniprep Kit (Epoch) for alkaline lysis and silica spin column chromatography, purified plasmid DNA was eluted with 50 μl nuclease-free water.

Dideoxy chain termination sequencing was performed, and the products separated on an Applied Biosystems 3730xl DNA Analyzer by the University of Cambridge Sequencing Facility. Sequencing results were visually inspected using CLC DNA workbench (CLC Bio). Multiple sequence alignment for FLRs was performed using Clustal X_2.0 and CLC DNA workbench, visualized using dendroscope (http://dendroscope.org/). Multiple sequence alignment for sequences from the experimental lines and from standard haplotypes was performed using MAFFT and trees displayed using Archaeopteryx.js (https://mafft.cbrc.jp/alignment/server/). A new BF2 sequence from the Sr1 haplotype found in one experimental line from France was deposited in GenBank with accession number MN103189.

## Results

### Design of a chicken MHC typing procedure by RSCA

**Fig. 1:**
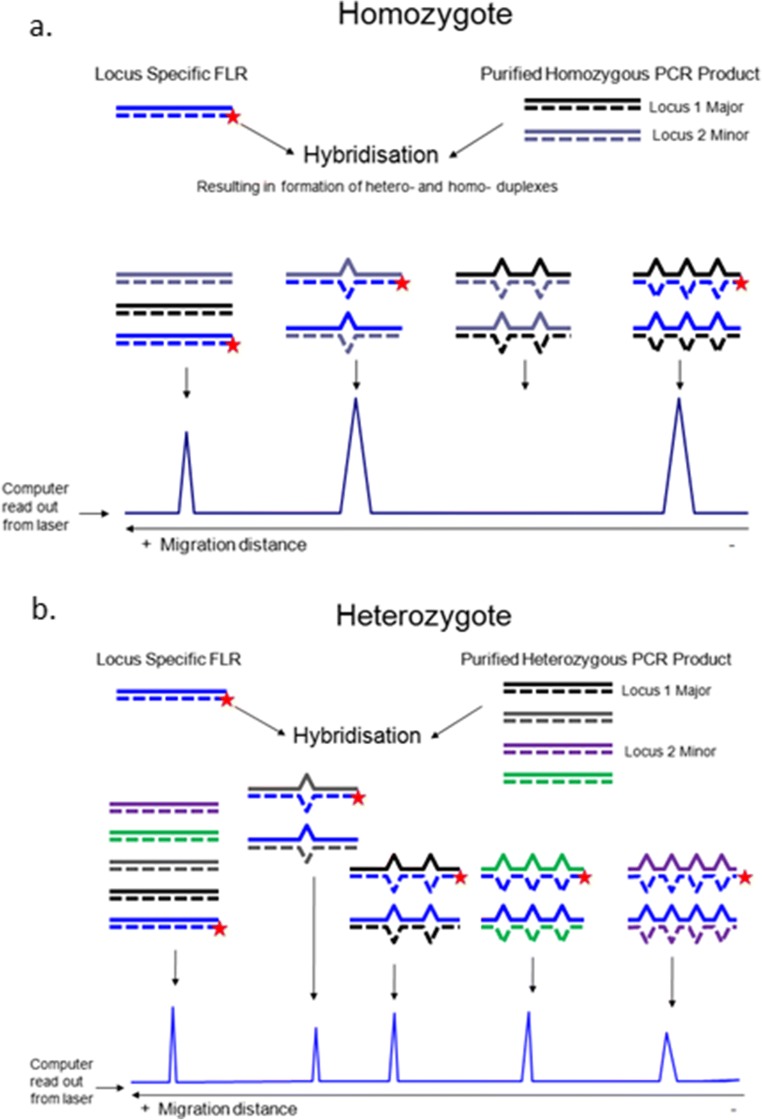
A diagram representing the four steps of RSCA (amplification to make an FLR, amplification from an experimental sample, hybridization to create DNA duplexes and analysis by capillary electrophoresis) with an idealized trace for a two gene system from **a** a homozygote (with all possible DNA duplexes shown, including those without an FLR, which would not be detected) and **b** a heterozygote (with only the DNA duplexes that include an FLR for detection shown). A homozygote sample produces one homoduplex and two heteroduplex peaks in the trace due to the labelled FLR; unlabelled strands form heteroduplexes but are not detected by the machine. A heterozygote sample produces one homoduplex and up to four heteroduplex peaks for a two-locus gene family (BLB or BF), but duplexes between unlabelled DNA strands are not detected (not shown for heterozygote). This figure is based conceptually on Argüello et al 1995

The RSCA method is based on hybridization of DNA fragments amplified by polymerase chain reaction (PCR) followed by analytical separation (Fig. 1). One fragment is amplified by particular primers from a known cloned sequence of which one primer is linked to a fluorescent molecule: the FLR. The other fragments are amplified using the same primers from an experimental sample. The FLR and the fragments amplified from the experimental sample are mixed, heated and allowed to cool, forming duplexes that can be separated by a variety of chromatography and/or electrophoresis methods. At the time that the work described in this report began (early 2010), the obvious separation technique based on ease and through-put was capillary electrophoresis using a so-called Genetic Analyzer; these instruments (or ones very much like them) are still commonly used to separate strands produced by dideoxy chain-termination sequencing (also known as Sanger sequencing) under denaturing conditions, but RSCA requires non-denaturing conditions. The output of the genetic analyzer is a trace (chromatogram or electrophoretogram), which for RSCA has a peak representing the homoduplex appearing first and other peaks with mismatches appearing later.

**Fig. 2:**
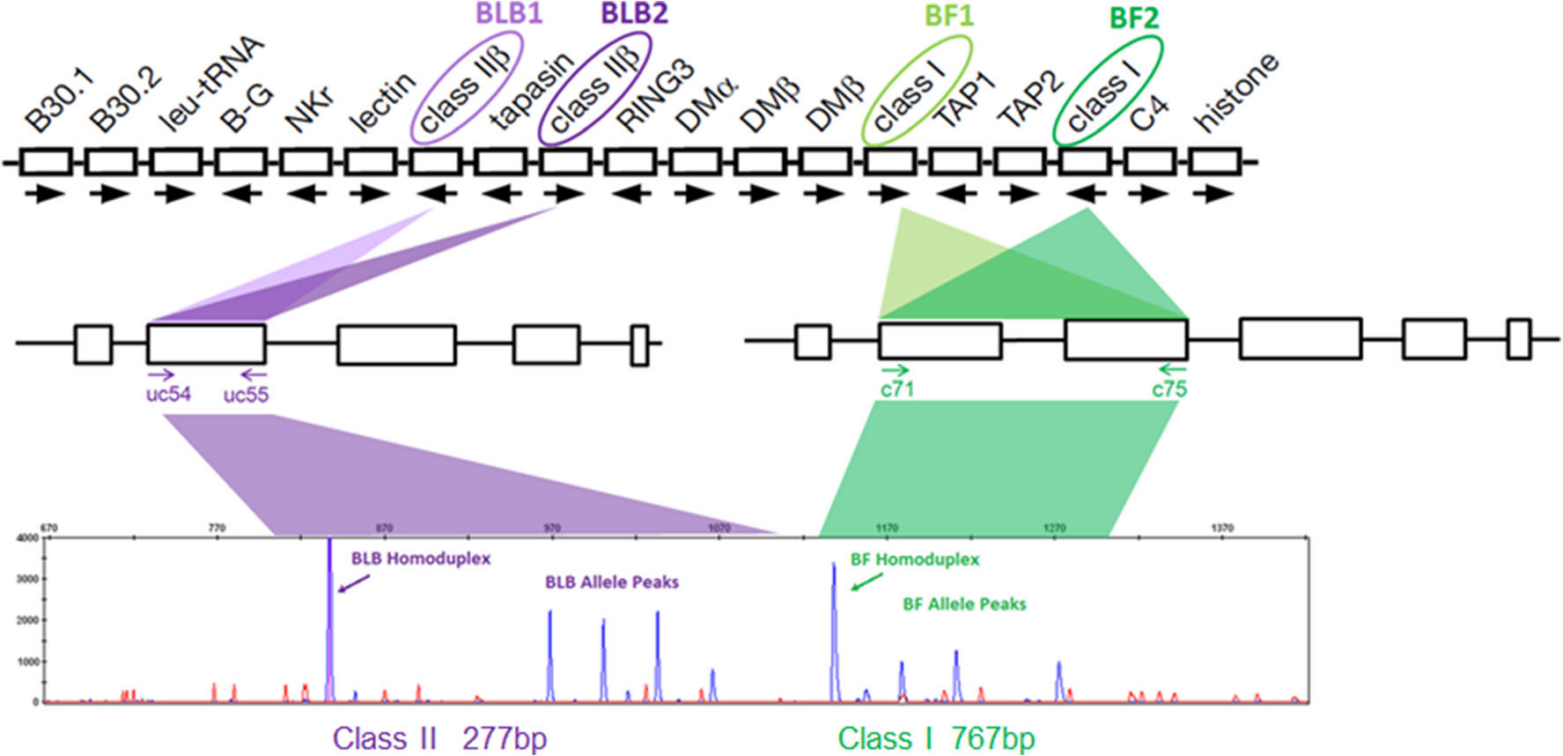
The genomic basis for simple RSCA typing of chicken classical class I and class II B genes. Top portion shows the BF-BL region, which contains only two BLB and two BF genes (based on Kaufman et al. 1999); middle portion shows intron-exon structures with the location of the primers (identified with local primer names) to amplify the exons encoding peptide-binding domains (Jacob et al. 2000; Shaw et al. 2007); bottom portion shows the RSCA trace for a heterozygote sample, with homo- and heteroduplexes indicated

There are several advantages in typing chicken classical MHC genes compared with many other species (Fig. 2). The patterns should be relatively simple, and copy number variation (CNV) is not a common feature (Kaufman et al. 1999; Hosomichi et al. 2008). There are only two classical class II B (β or light chain) genes for which the exon 2 sequence (which contains the polymorphic positions corresponding to the peptide-binding region) of both loci and all known alleles can be amplified by well-characterized primers to give a 277 bp fragment (including the primer sequences) (Jacob et al. 2000; Worley et al. 2008). There is virtually no polymorphism for the class II A (α or heavy chain) gene (Salomonsen et al. 2003), which can therefore be ignored. There are also only two classical class I (α or heavy chain) genes in chickens for which the region from exon 2 to exon 3 (which contain the polymorphic positions corresponding to the peptide-binding region formed by the α1 and α2 domains) of both loci and all known alleles can be amplified by well-characterized primers to give a fragment of only 767 bp (including the primer sequences) since the intron in between exon 2 and exon 3 is both short and highly conserved (Shaw et al. 2007). The choice of these primers means that the class II B DNA duplexes would appear earlier in the trace than the class I DNA duplexes, allowing a single combined run per sample. Finally, there are well-characterized chicken MHC haplotypes with known genomic sequences already cloned (Jacob et al. 2000; Shaw et al. 2007), allowing a choice of several FLRs in different runs to give the best chance at getting patterns with well-separated peaks.

**Fig. 3:**
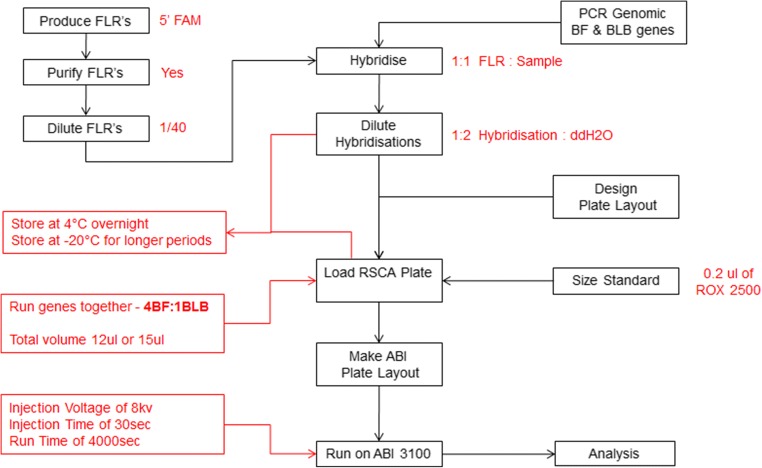
Work flow for the RSCA experiments described in this report. Each step of the flowchart (as well as several others not shown, some of which are discussed in the text) needed optimization in order to apply the typing method to unknown populations. Red text and boxes indicate the results of optimization steps

**Fig. 4:**
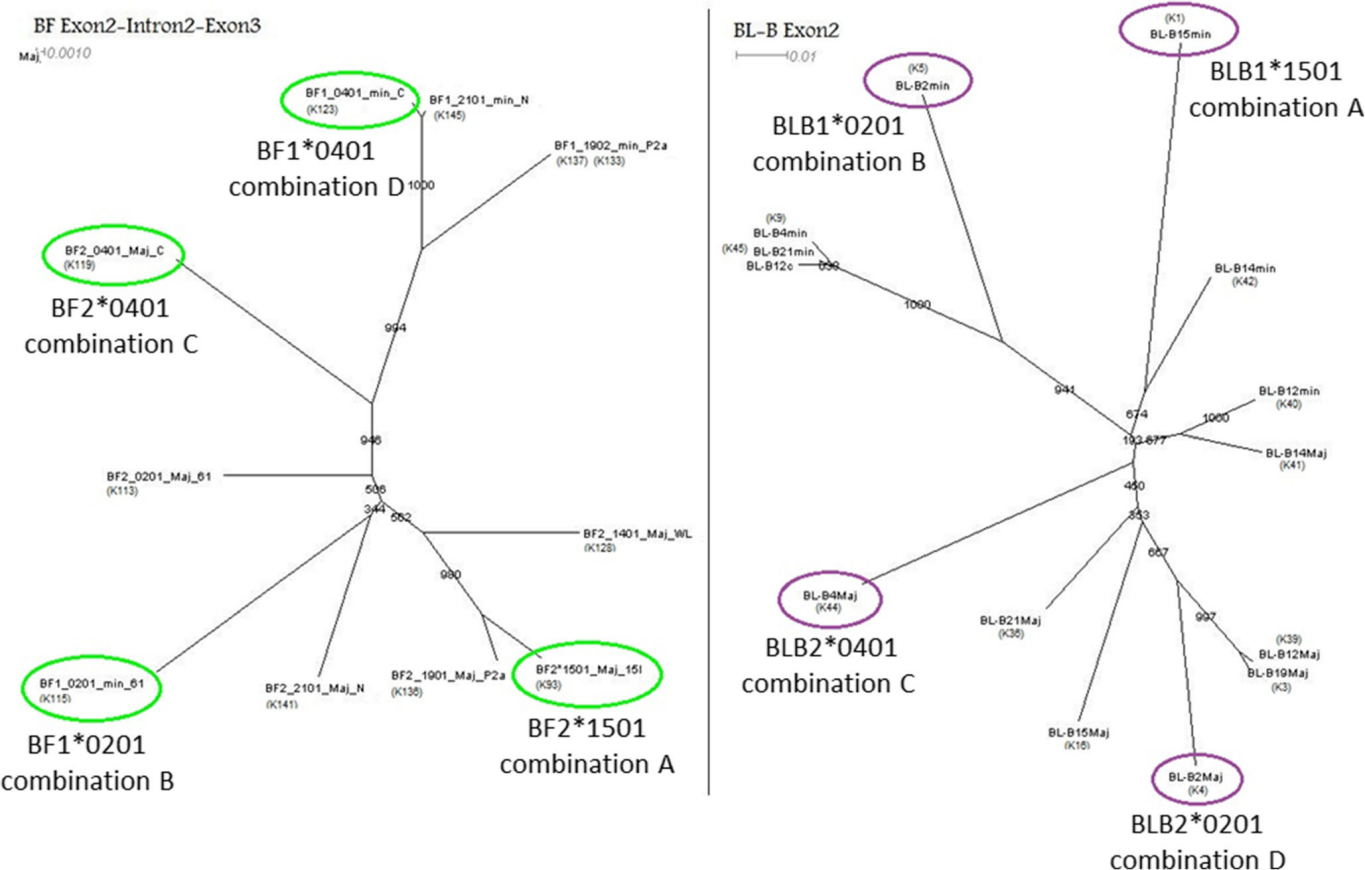
For both class I and class II B gene sequences, the four most divergent sequences were chosen to make FLRs. Nucleotide sequence alignments of exon 2 to exon 3 from class I (BF1 and BF2) genes (left panel) and of exon 2 from class II B (BLB1 and BLB2) genes (right panel) were used to construct neighbor-joining (NJ) trees, which were viewed using dendroscope. Four of the most divergent sequences are highlighted by green circles for class I, purple circles for class II B, and labelled for the four FLR combinations

**Fig. 5:**
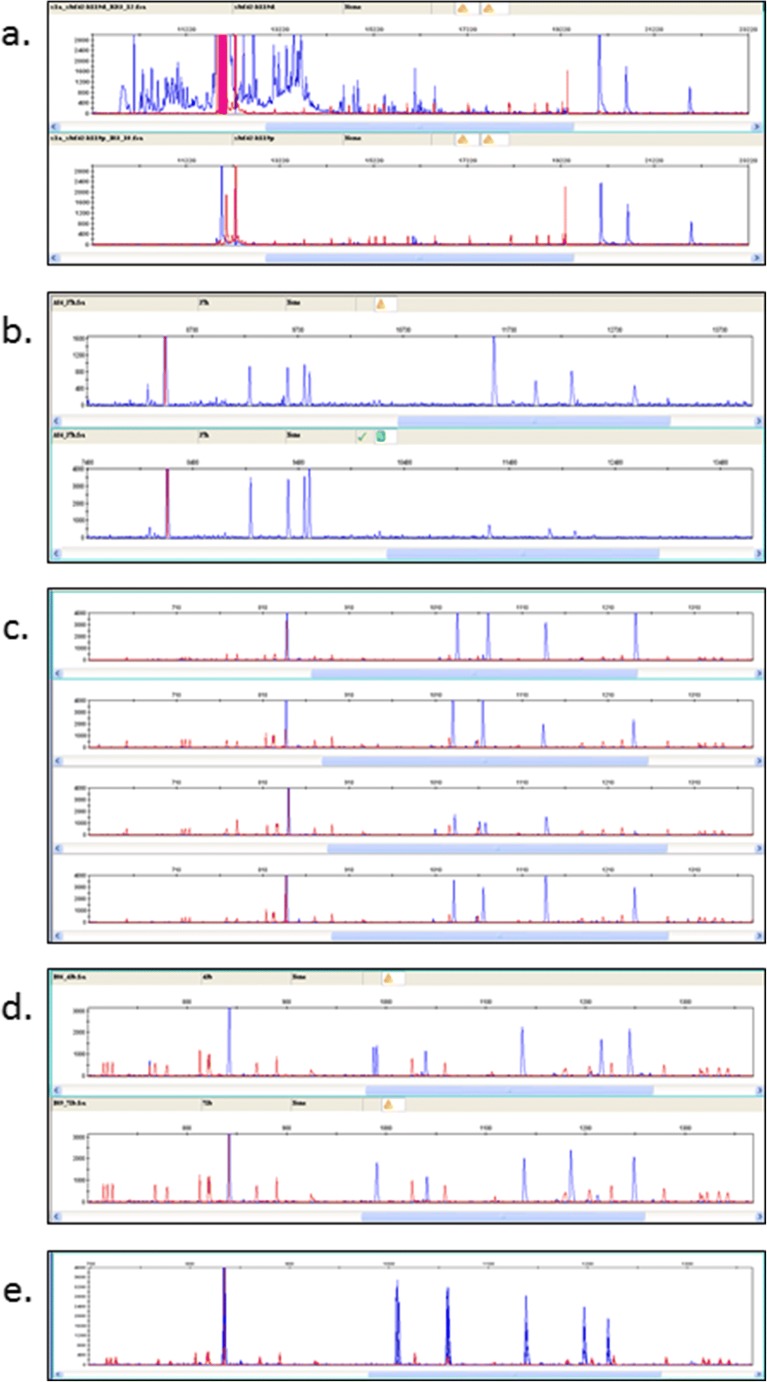
Several steps of the RSCA procedure needed optimization of which five are shown. All panels are RSCA traces from GenMapper software, with red size standard peaks (ROX2500 except for Fig. 5a) and blue fluorescent peaks from the hybridization mix. **a** Comparison after hybridization with a purified (top panel) and an unpurified (bottom panel) FLR. In this experiment, a BF2*0401 FLR preparation (before and after purification by silica spin column) was hybridized with an experimental B12 homozygote sample (blue peaks) and subjected to capillary electrophoresis along with ROX500 size standards (red peaks). This experiment established that the FLR needed to be purified before use and also that ROX2500 size standards were needed to cover the class I peaks. **b** Comparison after loading with hybridization mixtures of class II B mixture to class I mixture v/v 1:4 (top panel) and 1:1 (bottom panel). FLRs from BLB1*1401 and BF2*0401 were hybridized to DNA from a B4/B12 heterozygote sample. Increasing the volume of class I mix compared with class II B mix allowed the class I peaks to be detected because the concentrations were based on mass rather than molarity. **c** Comparison after storage of the hybridization mix and assembled plate under different conditions. An FLR produced from BF1*0201 was hybridized with genomic DNA from a B4/B12 heterozygote sample followed by storage under different conditions: hybridization not stored but assembled plate stored at − 20 °C for 5 days (top panel), hybridization stored at 4 °C for 10 days and assembled plate stored overnight at 4 °C (panel second from top), hybridization stored at 4 °C for 10 days and assembled plate stored at 4 °C for 5 days (panel third from top) and assembled plate stored at 4 °C overnight (bottom panel). This experiment shows that long-term storage of the assembled plate at 4 °C is not optimal. **d** Comparison of hybridization mixes assembled in the plate with different dilutions. FLRs from BLB1*0201 and BF1*0201 were hybridized to DNA from a B12 homozygote sample and assembled to give 12 μl in a well (top panel) and with a B19 homozygote sample and assembled with added water (to prevent too much evaporation during the run) giving 15 μl in a well (bottom panel). The comparable heights of the peaks show that the added water did not detract from the quality of the analysis. **e** Comparison of four identical samples overlaid to show reproducibility within a run. FLRs from BLB2*0401 and BF2*0401 were hybridized to DNA amplified from a B2 homozygote sample

**Fig. 6:**
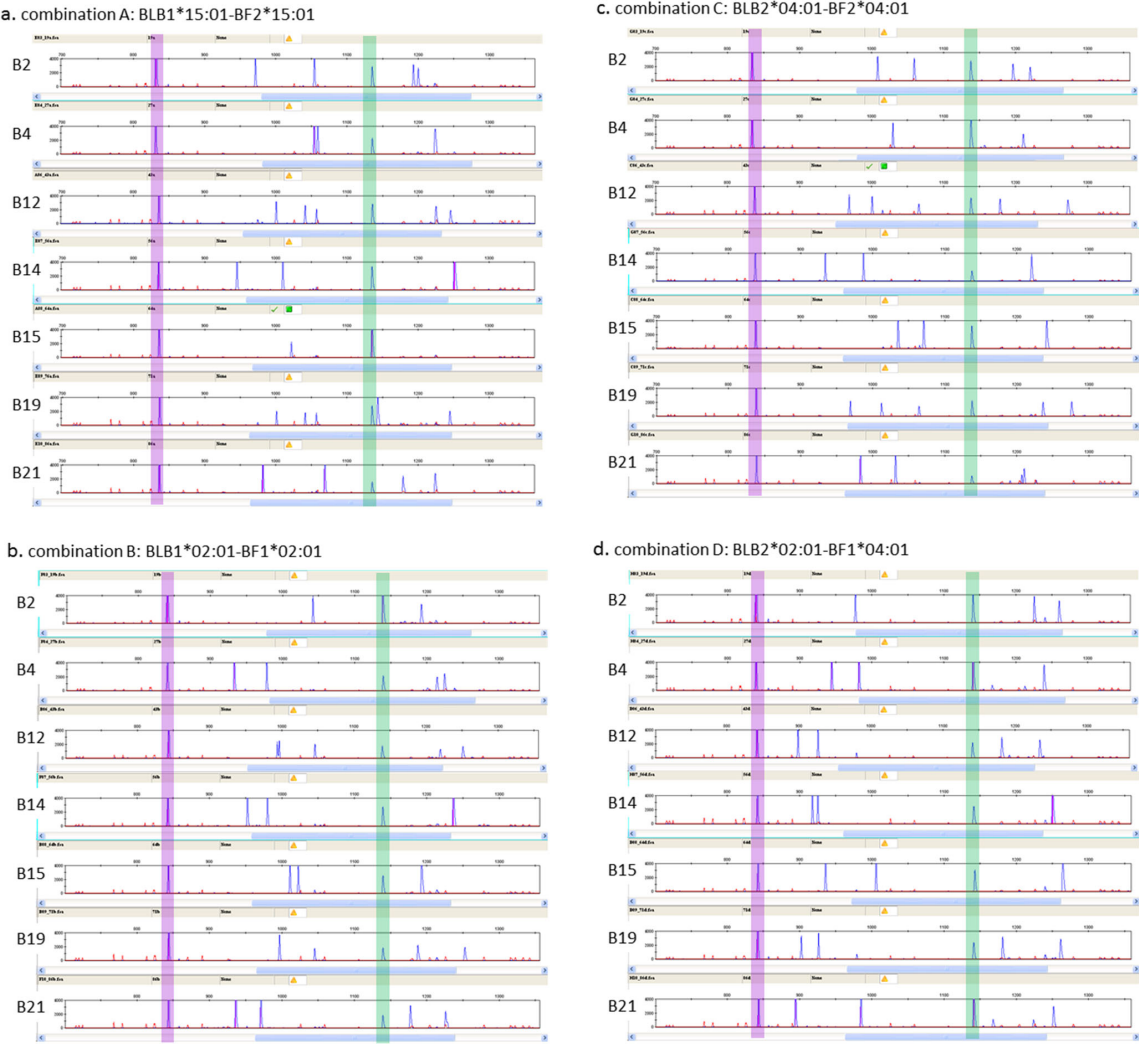
Examples of RSCA traces from hybridization of genomic DNA from homozygous samples of haplotype B2, B4, B12, B14, B15, B19 and B21 (from top to bottom) with **a** FLR combination A (BLB1*1501 and BF2*1501), **b** FLR combination B (BLB1*0201 and BF1*0201), **c** FLR combination C (BLB2*0401 and BF2*0401) and **d** FLR combination D (BLB2*0201 and BF1*0401). Peaks of size standard are red, while peaks of fluorescence from FLRs are blue, with migration scores of peaks determined with reference to the size standards by the GeneMapper software. Homoduplexes are highlighted, purple for class II B peaks and green for class I peaks

**Fig. 7:**
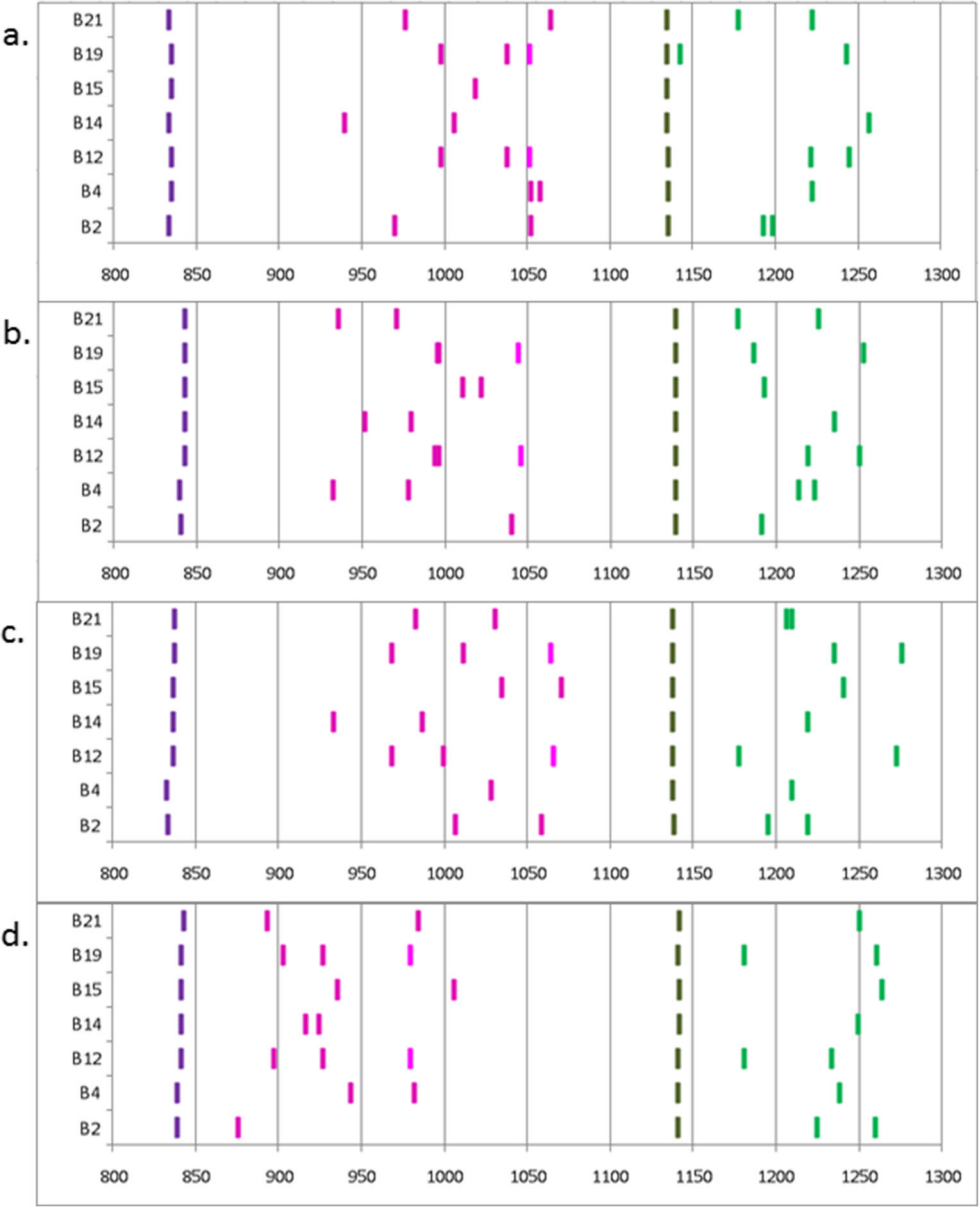
Chart of migration scores for peaks from samples of haplotype B2, B4, B12, B14, B15, B19 and B21 (from bottom to top) with **a** FLR combination A (BLB1*1501 and BF2*1501), **b** FLR combination B (BLB1*0201 and BF1*0201), **c** FLR combination C (BLB2*0401 and BF2*0401) and **d** FLR combination D (BLB2*0201 and BF1*0401) based on data in Fig. 6a–d. The migration scores are indicated by lines that are purple for homoduplex peaks and pink for experimental peaks from class II FLRs and dark green for homoduplex peaks and light green for experimental peaks from class I FLRs

**Fig. 8:**
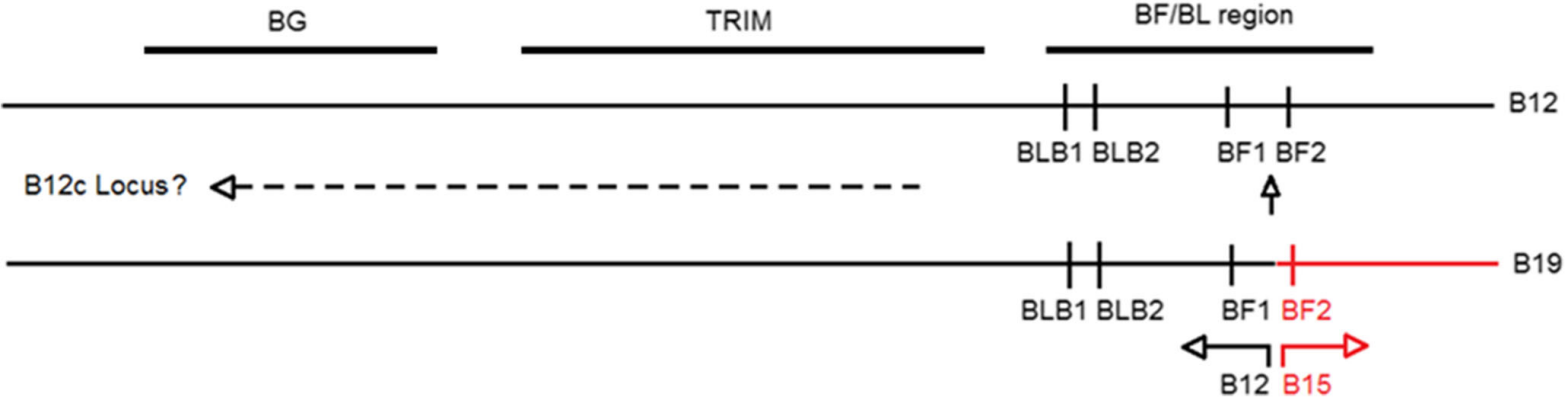
Inferred location of the third BLB gene by comparison of B12 and B19 haplotypes for the BG, TRIM and BF-BL regions, showing the recombination between the B12 and B15 haplotypes to give the B19 haplotype. Black horizontal line indicates sequence identical (or nearly so) with the B12 haplotype, red horizontal line indicates sequence nearly identical to the B15 haplotype, vertical arrow indicates recombination event between BF1 and BF2 and dashed line indicates the likely location of the B12c gene given the data in this report

**Fig. 9:**
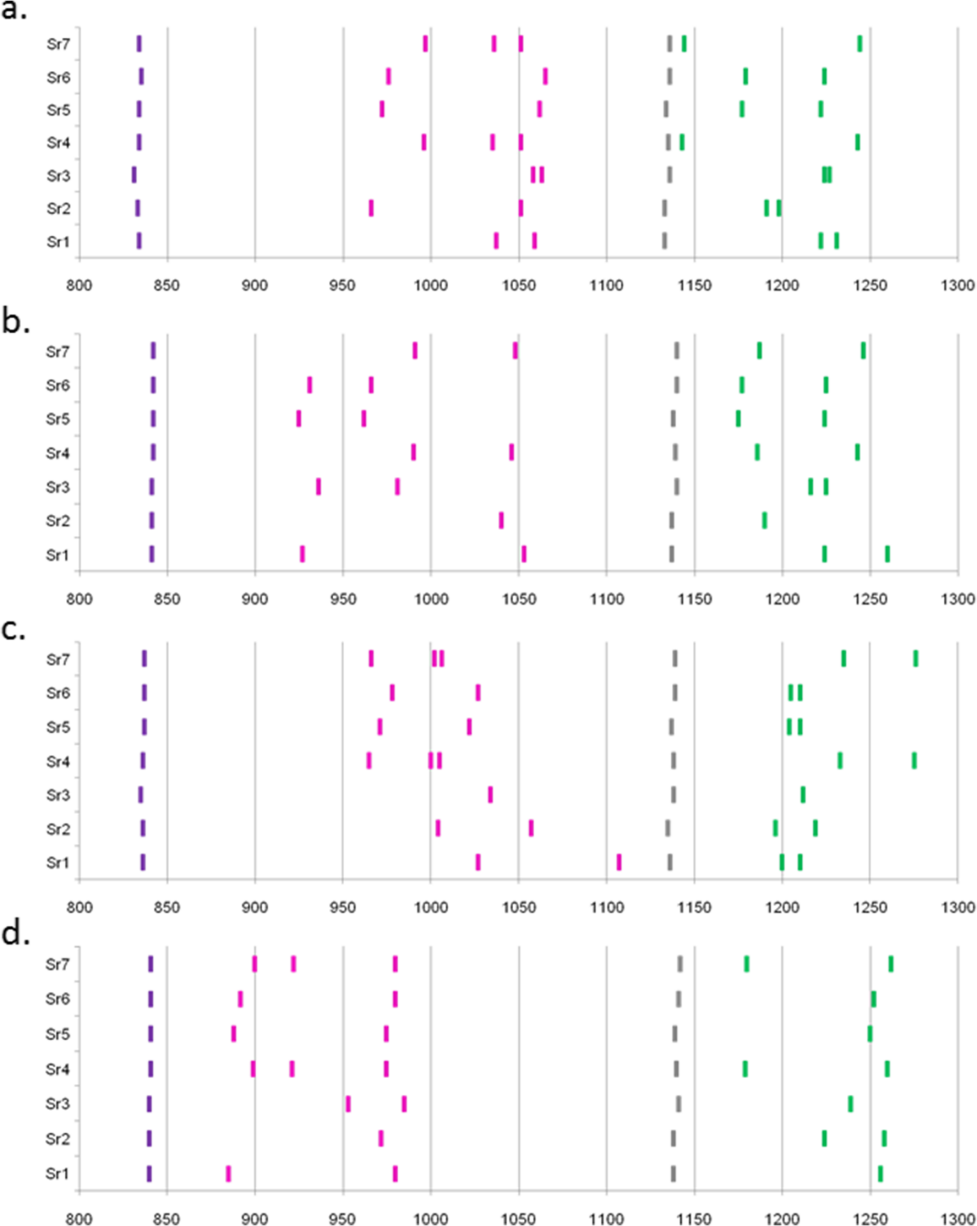
Chart of migration scores for peaks from samples of French experimental lines corresponding to haplotypes Sr1, Sr2, Sr3, Sr4, Sr5, Sr6 and Sr7 (from bottom to top) with **a** FLR combination A (BLB1*1501 and BF2*1501), **b** FLR combination B (BLB1*0201 and BF1*0201), **c** FLR combination C (BLB2*0401 and BF2*0401) and **d** FLR combination D (BLB2*0201 and BF1*0401). The migration scores are indicated by lines that are purple for homoduplex peaks and pink for experimental peaks from class II FLRs, and dark green for homoduplex peaks and light green for experimental peaks from class I FLRs

**Fig. 10:**
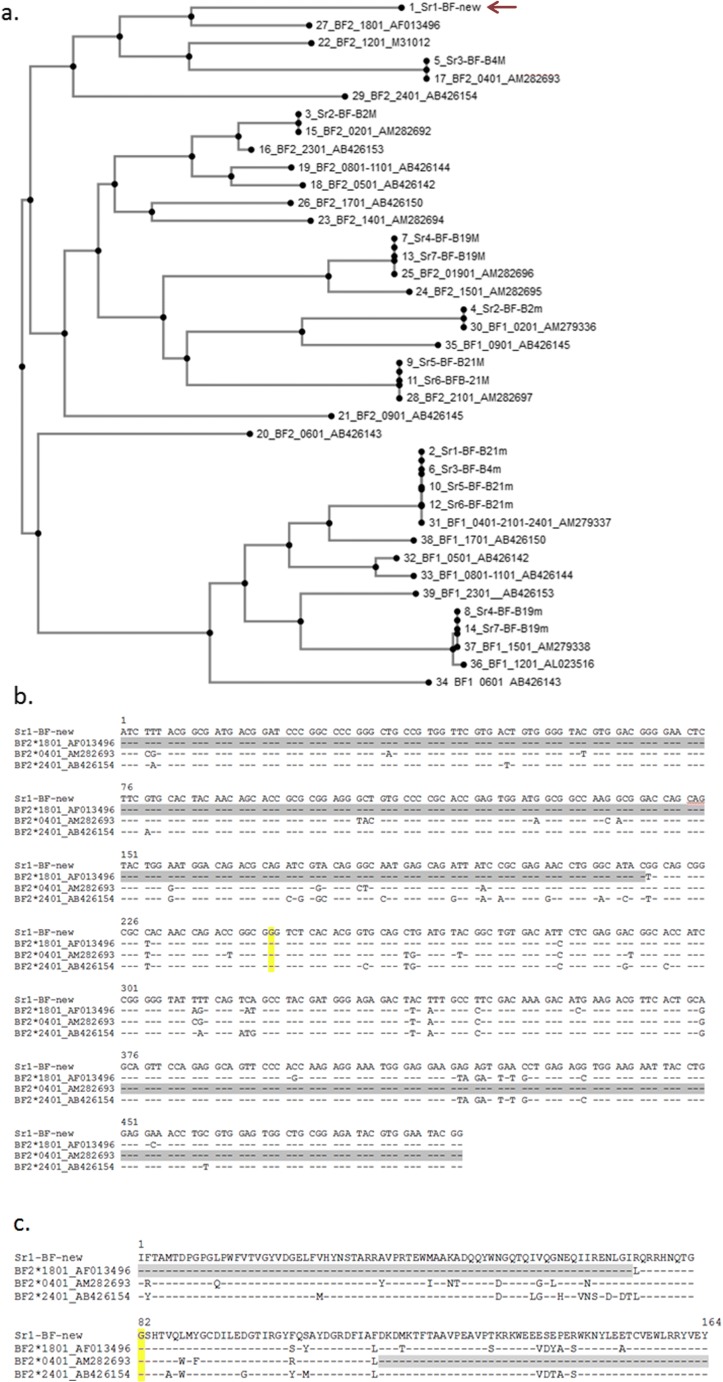
The BF alleles from the French experimental lines are identical with alleles of standard haplotypes (at least in the region amplified by the primers), except for one previously unreported BF2 allele from the Sr1 haplotype. **a** neighbor-joining tree of nucleotide sequences (as amplified by the primers, but without the primer sequences or the introns) from standard haplotypes [(named as per current nomenclature (Miller et al. 2004)], followed by an accession number for a representative sequence, and from the haplotypes from the French experimental lines [names have a number from the MAFFT alignment, followed by the Sr haplotype number, followed by the identification using an older nomenclature (Wallny et al. 2006)]. Sequences are identical when a vertical line links them. Red arrow indicates the new BF2 allele from the recombinant Sr1 haplotype, which is most closely related to BF2*1801 and far away from BF2*2401. **b** and **c** Nucleotide and amino acid sequence alignments of the new class I allele of the Sr1 haplotype with BF2*1801, BF2*0401 and BF2*2401 [all as amplified by the primers, but without the primer sequences or the introns, (Hosomichi et al. 2008)], with long stretches of identical sequence highlighted in grey and with the beginning of exon 3 highlighted in yellow. The BF2 sequence from Sr1 differs from BF2*2401 throughout the amplified sequence but is identical with the first 217 nucleotides (72 amino acids) of the amplified sequence of BF2*1801 from exon 2 and with the last 119 nucleotides (48 amino acids) of the amplified sequence of BF2*0401 from exon 3. The new BF2 sequence from the Sr1 haplotype was deposited in GenBank with accession number MN103189

### Optimization of a chicken MHC typing procedure by RSCA

There are many steps to the RSCA procedure (Fig. 3), and reliable high throughput analysis required these steps to be optimized. The steps optimized included the choice, amplification conditions and the purification of the FLRs before use, the amplification conditions for the experimental samples, the choice and amount of the fluorescent standards to be loaded, the storage conditions of samples before loading, the relative amounts of the class I and class II experimental amplicons to be loaded, the volume of the solution in the plates to be loaded and the electrophoretic conditions of the chromatographic separation; all of which are described in detail in a thesis (Potts 2016). The final protocol is presented in the Materials and Methods, but the optimization of some steps deserves further mention.

FLRs from both class I and class II B genes were required and, because sequences identical to the FLRs produce homoduplexes and thus cannot be typed, more than one of each was required. The class I and class II B genes from seven MHC haplotypes had been cloned and characterized in detail (Jacob et al. 2000; Shaw et al. 2007). The sequences were compared by alignment and phylogenetic analysis (Fig. 4) and 14 FLRs were produced and tested in pilot runs (data not shown). The most disparate sequences by phylogenetic analysis were found to give good separations in the pilot runs, and so four combinations of these class I and class II B genes, named A, B, C and D, were chosen for FLRs. However, it was important to purify the FLRs before use (Fig. 5a).

It was realized early on that the size difference of the amplicons from class I and class II B might allow the class II B peaks to appear well before the class I peaks in the electrophoretic chromatography. This was a significant advantage since the class II B and class I hybridization samples could be combined in one run so that, overall, only half as many runs would be required. However, trial runs showed that the ratio of class II B and class I hybridization products was critical to achieving similar peak amplitudes. Presumably this is due to the fact that the concentrations used were based on mass rather than number of molecules, but the length (and thus the mass) of BF molecules was greater than for BLB molecules. Therefore, a much greater concentration based on mass (for instance, ng/μl) of the BF duplexes compared to BLB duplexes would be required to give a similar concentration based on number of molecules (for instance, pmoles/μl), given that there would be only one FLR per molecule. A ratio of one volume of class II B hybridization product to four volumes of class I hybridization product yielded similar sized peaks across the chromatogram (Fig. 5b). In this trace from an MHC heterozygote, a peak for the class II B homoduplex is followed by four peaks representing the class II B amplicons from the experimental sample, then the class I homoduplex followed by three peaks representing the class I amplicons from the experimental sample.

Amount and storage of the samples after hybridization were important considerations. Although the capillary electrophoresis instruments were widely available at the time of this work, most were used under denaturing conditions for resolution of DNA fragments after Sanger sequencing. Access to analysis under non-denaturing conditions was very limited due to the perception that changing the buffers back and forth caused more rapid degradation of the columns. It was found that storing the hybridized samples for more than 24 h at 4 °C led to poor separation, but that freezing samples at − 20 °C over considerable periods of time was not detrimental (Fig. 5c). The long periods of time required to analyze all the samples led to evaporation of liquid from wells, and it was found that diluting hybridized samples up to 15 μl gave the same results as with the normal volume but without loss of samples from dried out wells (Fig. 5d).

After all the steps had been optimized, the procedure gave reproducible results within experiments. However, two problems were never fully solved. First, fluorescent standards were added to each sample to establish standard positions on the trace, but there were no fluorescent standards commercially available that were dedicated to analysis under non-denaturing conditions. Various standards designed for denaturing conditions were trialed and the ROX2500 chosen, but the detection software often missed one or more of the standard peaks, which had to be added to the analysis manually. Second, the location of the sample peaks within the trace could be highly reproducible within runs (Fig. 5e), but, over the course of many experiments, the positions of sample peaks with respect to standard peaks would drift. A program to allow for such drift (“the App”) was trialed but not extensively implemented (Potts 2016).

### Validation of a chicken MHC typing procedure by RSCA

Several experimental chicken lines have been widely used over the last 60 years, mostly bearing one of the so-called “standard B haplotypes,” some of which have been extensively characterized genetically, structurally and functionally (Miller et al. 2004). Nine of these experimental lines bearing seven well-characterized standard haplotypes were available to use for validation (Jacob et al. 2000; Shaw et al. 2007). The RSCA typing procedure was carried out on multiple samples from each of the nine lines using all four FLR combinations, of which examples are shown (Fig. 6) and are summarized in a diagram of migration scores calculated by the commercial GeneMapper software (Fig. 7).

Clear peak patterns are evident, starting with the class II B homoduplex followed by the experimental class II B peaks and then the class I homoduplex followed by the experimental class I peaks. Most of the samples show two experimental class II B peaks and two experimental class I peaks, as would be expected for two class II B genes and two class I genes in a homozygote animal.

However, fewer peaks were found in those runs for which one of the genes in the experimental sample was identical to one of the FLRs. FLR combination B consisted of BLB1*0201 and BF1*0201, so one each of the expected class II B and class I peaks would have been present in the homoduplexes for a B2 experimental sample, yielding only one experimental class II B peak and one experimental class I peak, as is in fact seen (Fig. 6b, first panel). Similarly, FLR combination C consisted of BLB2*0401 and BF2*0401, so only one experimental class II B peak and one experimental class I peak was found for the B4 sample with this FLR (Fig. 6c, second panel). Finally, FLR combination D consisted of BLB2*0201 and BF1*0401, so compared with general expectations, with this FLR, one less experimental class II B peak was found in the B2 sample (Fig. 6d, first panel) and one less experimental class I peak was found in the B4 sample (Fig. 6d, second panel) but also in the B21 sample (Fig. 6d, seventh panel), the latter because the BF1 sequences from B4 and B21 are identical from exon 2 to exon 3 (Wallny et al. 2006; Shaw et al. 2007).

The same logic would lead to the expectation that FLR combination A, composed of BLB1*1501 and BF2*1501, would lead to one less class II B peak and one less class I peak than expected in the B15 sample, but, in fact, no experimental class I peaks were found (Fig. 6a, fifth panel). However, this is easily explained, as it is known that the BF1 genes are pseudogenes in the B14 and B15 haplotypes and cannot be amplified with these primers (Shaw et al. 2007). B14 samples also show only one experimental class I peak with FLR combination A (Fig. 6a, fourth panel), while both B14 and B15 samples show only one experimental class I peak with other three FLR combinations (Fig. 6b–d, fourth and fifth panels). It was initially a surprise that only one experimental class I peak was found for B4 with FLR combination A (Fig. 6a, second panel), but it became apparent throughout many runs that this peak was fatter than most other peaks and is composed of two peaks that are nearly superimposed.

One extra experimental class II B peak compared to initial expectations was found for three FLRs in the B12 sample, although the third peak ran very close to another peak with FLR combination B and had a very low amplitude with FLR combination D (Fig. 6a–d, third panels). This result fits with the previous reports of a third BLB locus (so-called B12c) in this haplotype (Zoorob et al. 1993; Jacob et al. 2000). However, it was initially a surprise to discover a third class II B peak in the B19 samples, although the third peak co-migrated with another peak with FLR combination B (giving a peak with double the amplitude of the other peaks) and had a very low amplitude with FLR combination D (Fig. 6a–d, sixth panels). In fact, the B19 haplotype is known to be a recombinant between the B12 haplotype and the B15 haplotype, with the BLB1 and BLB2 genes from the B19 haplotype identical in sequence to those of the B12 haplotype. This finding suggests that the third locus is present on one side of the MHC (Fig. 8).

Interestingly, one of the two class I peaks in the B19 sample with FLR combination A was very close to the class I homoduplex (Fig. 6a, sixth panel). This presumably reflects the fact that the sequence of BF2*1901 in exon 2 to exon 3 differs from the FLR of BF2*1501 by only a few residues, while the other class I peak runs in the same position as one of the class I peaks in the B12 haplotype, likely to be BF1*1201, which is identical in sequence to BF1*1901 (Wallny et al. 2006; Shaw et al. 2007).

Overall, the results fit with everything known or suspected about the gene content and sequence in these standard haplotypes, allowing this RSCA system for chicken MHC genes to be used with confidence. However, the fact that fewer peaks were found with some experimental samples with some FLR combinations underscores the fact that multiple FLRs are needed to ensure that all the sequences present lead to detectable peaks.

### Determination of the MHC genes present in five chicken lines from France

In order to test the RSCA typing system on truly unknown samples, 100 samples of genomic DNA from the INRA Unité PFIE chicken facility in Nouzilly were analyzed. The samples were first analyzed by RSCA using the four FLR combinations, and then selected samples were amplified, cloned and sequenced for a final identification.

Seven patterns were initially identified by RSCA using all four FLR combinations and named Sr1–Sr7, although extremely similar patterns were found for each of two pairs of patterns (Fig. 9). Subsequent cloning and sequencing revealed ten class II B and nine class I sequences, all but one of which are identical to a known sequence by phylogenetic analysis (Fig. 10a). These sequences were found in five haplotypes, four of which appear to be standard: Sr2 is B2; Sr3 is B4; Sr4 and Sr7 are both B19; and Sr5 and Sr6 are both B21.

In contrast, Sr1 is new haplotype that has BLB1, BLB2 and BF1 from the standard B24 haplotype, but BF2 with a previously unreported sequence. Based on the phylogenetic analysis (Fig. 10a), the BF2 sequence from Sr1 is most closely related to BF2*1801, which initially suggested the possibility that the Sr1 haplotype was a recombinant between the B24 and B18 haplotypes, with subsequent changes to the BF2 gene. Such a scenario is much like that postulated for the appearance of the B19 haplotype (Fig. 8).

However, nucleotide and amino acid sequence alignments suggest a more complex history of the Sr1 haplotype. The BF2 sequence from Sr1 (determined by multiple clones from several samples) has many differences compared with BF2*2401, the sequence expected from the B24 haplotype, but has long stretches of sequence identical to BF2*1801 in exon 2 and to BF2*0401 in exon 3 (Fig. 10b and c). Multiple recombinational events (for instance, gene conversion) may have contributed to the appearance of the BF2 gene from Sr1. Alternatively, the Sr1 haplotype could be ancestral, with the B18 haplotype arising by a single recombination event. Such recombination fits well with reports of recombination within the BF-BL region as revealed by SNP typing (Fulton et al. 2016b).

Subsequent disclosure revealed that the samples originated from five experimental lines kept at the INRA Unité PFIE chicken facility, each with 20 samples. The samples from lines named B13, B19 and B21 had only homozygotes of B4 (Sr3), B19 (Sr4) and B21 (Sr5) haplotypes, respectively. The B13 haplotype is nearly identical with B4 in the BF-BL region that encodes the classical class I and class II B genes, but is different in the BG region that generally determines the serological identification (Miller et al. 2004). In fact, a single nucleotide difference in the intron between exon 2 and exon 3 of BF2 was found that could distinguish B4 from B13, in agreement with alignments based on published sequences (Shaw et al. 2007; Hosomichi et al. 2008). The samples from line PA12 contained both homozygotes and heterozygotes of B19 (Sr7) and B21 (Sr6) haplotypes, while the samples from line LD had both homozygotes and heterozygotes of the unique Sr1 haplotype and the B2 (Sr2) haplotype.

## Discussion

MHC genes comprise the most polymorphic loci known in genetics, with over 10,000 alleles of classical class I and class II genes among the roughly 7.5 billion people currently on our planet. In comparison, only some 50 alleles have been reliably described from the estimated 80 billion chickens on the planet each year. Given that chickens suffer from many economically important diseases (some of which, like influenza, are zoonotic) and that MHC alleles are thought to be primarily selected by arms races with pathogens, one might expect many more alleles to be found. To our knowledge, there has been no systematic analysis of polymorphism of the alleles of chicken MHC genes at a large scale. The method and results in this report describe the first steps in a long-term project that has already identified hundreds of alleles in several dozen haplotypes over the last 10 years (C. Tregaskes, R. Martin, F. Coulter, E. Doran and J. Kaufman, unpublished). The results of all the large-scale screening deserve publications of their own, but this report is the fundamental basis for them all.

Hybridization methods were originally developed to type human MHC genes and have been widely used to type MHC alleles, although they are being replaced by SNP typing and/or direct next generation sequencing (NGS). We have used RSCA successfully to type red junglefowl and commercial populations, not just for MHC (Worley et al. 2008; Potts 2016) but also for chicken immunoglobulin-like receptors (CHIR-AB1) genes (Meziane et al. 2019). However, there are various disadvantages to typing by RSCA. First, the procedure has required significant optimization of many of the steps and is relatively cumbersome to perform. Second, reproducibility within runs is excellent but between runs over time is not perfect; the peak positions relative to the fluorescent standard drifted between runs and the amplitudes of the peaks varied somewhat. Third, additional cloning and sequencing was necessary to identify the exact alleles after screening by RSCA. So, we now have developed a PCR-NGS typing system to continue this project (C. Tregaskes, R. Martin, F. Coulter, E. Doran and J. Kaufman, unpublished).

However, we have found significant advantages of RSCA compared with direct sequencing, including relative insensitivity to low-level contamination and far less identification of chimeric sequences from re-priming by partial amplicons (Potts 2016). The reason for both of these advantages is probably that the peak amplitude reflects the number of identical sequences produced during amplification, and so PCR errors must happen very early or the incorrect sequences must amplify extremely efficiently compared with the real sequences in order to produce a credible peak on the trace. As a third advantage, we were able to multiplex the analysis by ensuring that the amplicon sizes were sufficiently different. Finally, although we used rather sophisticated and well-engineered capillary electrophoresis instruments to detect the DNA duplexes after hybridization, relatively low tech separation systems such as gel electrophoresis can also be used (Chavatte-Palmer et al. 2007; Babik 2010). So, RSCA may remain a technique with some use in the future.

In this report, we describe in some detail the optimization and validation of the RSCA approach to typing chicken MHC genes, which provides the basis for large scale typing of the chicken MHC. Such typing provides a foundation for simpler approaches in the future since particular alleles and haplotypes can be correlated with the growing body of microsatellite and SNP data (Iglesias et al. 2019; Fulton et al. 2006, 2016a, 2016b, 2017; McElroy et al. 2005; Nguyen-Phuc et al. 2016), eventually allowing imputation of alleles from flanking SNPs, as is routinely done for the human MHC. In addition, we make two discoveries. We describe for the first time the presence of a third BLB gene in the B19 haplotype, suggesting strongly that this gene is present in B12 and B19 haplotypes outside the original B12 cosmid sequence (Kaufman et al. 1999) but on the side of the BG region. We also provide molecular identification of MHC haplotypes in French experimental chicken lines that were previously known only by serology and define a new class I sequence from a new apparent recombinant haplotype. Such discoveries in new lines and populations, along with their correlation with phenotypes including disease resistance, are to be expected in the future.

## References

[CR1] Argüello JR, Little AM, Bohan E, Goldman JM, Marsh SG, Madrigal JA (1998). High resolution HLA class I typing by reference strand mediated conformation analysis (RSCA). Tissue Antigens.

[CR2] Atkinson D, Shaw I, Jacob J, Kaufman J (2001) DM gene polymorphisms: co-evolution or coincidence? In Proceedings of the Avian Immunology Research Group, 7-10 October 2000. Ithaca NY Edited by KA Schat:163–165

[CR3] Babik W (2010). Methods for MHC typing in non-model vertebrates. Mol Ecol Resour.

[CR4] Boonyanuwat K, Thummabutra S, Sookmanee N, Vatchavalkhu V, Siripholvat V (2006). Influences of major histocompatibility complex class I haplotypes on avian influenza virus disease traits in Thai indigenous chickens. Anim Sci J.

[CR5] Bryceson YT, Chiang SC, Darmanin S, Fauriat C, Schlums H, Theorell J, Wood SM (2011). Molecular mechanisms of natural killer cell activation. J Innate Immun.

[CR6] Cassidy SA, Cheent KS, Khakoo SI (2014). Effects of Peptide on NK cell-mediated MHC I recognition. Front Immunol.

[CR7] Cassidy S, Mukherjee S, Myint TM, Mbiribindi B, North H, Traherne J, Mulder A, Claas FH, Purbhoo MA, Das J, Khakoo SI (2015). Peptide selectivity discriminates NK cells from KIR2DL2- and KIR2DL3-positive individuals. Eur J Immunol.

[CR8] Chattaway J, Ramirez-Valdez RA, Chappell PE, Caesar JJ, Lea SM, Kaufman J (2016) Different modes of variation for each BG lineage suggest different functions. Open Biol 6 10.1098/rsob.160188 PMC5043582 27628321

[CR9] Chavatte-Palmer P, Guillomot M, Roïz J, Heyman Y, Laigre P, Servely JL, Constant F, Hue I, Ellis SA (2007). Placental expression of major histocompatibility complex class I in bovine somatic clones. Cloning Stem Cells.

[CR10] Chazara O, Tixier-Boichard M, Morin V, Zoorob R, Bed'hom B (2011). Organisation and diversity of the class II DM region of the chicken MHC. Mol Immunol.

[CR11] Dilthey AT, Moutsianas L, Leslie S, McVean G (2011). HLA*IMP--an integrated framework for imputing classical HLA alleles from SNP genotypes. Bioinformatics.

[CR12] Ewald SJ, Livant EJ (2004). Distinctive polymorphism of chicken B-FI (major histocompatibility complex class I) molecules. Poult Sci.

[CR13] Fulton JE, Juul-Madsen HR, Ashwell CM, McCarron AM, Arthur JA, O'Sullivan NP, Taylor RL (2006). Molecular genotype identification of the Gallus gallus major histocompatibility complex. Immunogenetics.

[CR14] Fulton JE, Lund AR, McCarron AM, Pinegar KN, Korver DR, Classen HL, Aggrey S, Utterbach C, Anthony NB, Berres ME (2016). MHC variability in heritage breeds of chickens. Poult Sci.

[CR15] Fulton JE, McCarron AM, Lund AR, Pinegar KN, Wolc A, Chazara O, Bed'Hom B, Berres M, Miller MM (2016). A high-density SNP panel reveals extensive diversity, frequent recombination and multiple recombination hotspots within the chicken major histocompatibility complex B region between BG2 and CD1A1. Genet Sel Evol.

[CR16] Fulton JE, Berres ME, Kantanen J, Honkatukia M (2017). MHC-B variability within the Finnish Landrace chicken conservation program. Poult Sci.

[CR17] Goto RM, Wang Y, Taylor RL, Wakenell PS, Hosomichi K, Shiina T, Blackmore CS, Briles WE, Miller MM (2009). BG1 has a major role in MHC-linked resistance to malignant lymphoma in the chicken. Proc Natl Acad Sci U S A.

[CR18] Hirschhorn JN, Gajdos ZK (2011). Genome-wide association studies: results from the first few years and potential implications for clinical medicine. Annu Rev Med.

[CR19] Hosomichi K, Miller MM, Goto RM, Wang Y, Suzuki S, Kulski JK, Nishibori M, Inoko H, Hanzawa K, Shiina T (2008). Contribution of mutation, recombination, and gene conversion to chicken MHC-B haplotype diversity. J Immunol.

[CR20] Iglesias GM, Canet ZE, Cantaro H, Miquel MC, Melo JE, Miller MM, Berres ME, Fulton JE (2019). Mhc-B haplotypes in "Campero-Inta" chicken synthetic line. Poult Sci.

[CR21] Jacob JP, Milne S, Beck S, Kaufman J (2000). The major and a minor class II beta-chain (B-LB) gene flank the Tapasin gene in the B-F /B-L region of the chicken major histocompatibility complex. Immunogenetics.

[CR22] Kaplan MH, Hufford MM, Olson MR (2015). The development and in vivo function of T helper 9 cells. Nat Rev Immunol.

[CR23] Kaufman J (2018). Unfinished business: evolution of the adaptive immune system of jawed vertebrates. Annu Rev Immunol.

[CR24] Kaufman J (2018). Generalists and specialists: a new view of how MHC class I molecules fight infectious pathogens. Trends Immunol.

[CR25] KAUFMAN JIM, VOLK HEINER, WALLNY HANS-JOACHIM (1995). A "Minimal Essential Mhc" and an "Unrecognized Mhc": Two Extremes in Selection for Polymorphism. Immunological Reviews.

[CR26] Kaufman J, Milne S, Göbel TW, Walker BA, Jacob JP, Auffray C, Zoorob R, Beck S (1999). The chicken B locus is a minimal essential major histocompatibility complex. Nature.

[CR27] Kim T, Hunt HD, Parcells MS, van Santen V, Ewald SJ (2018). Two class I genes of the chicken MHC have different functions: BF1 is recognized by NK cells while BF2 is recognized by CTLs. Immunogenetics.

[CR28] Klein J (1986). The Natural History of the Major Histocompatibility Complex.

[CR29] Lima-Rosa CA, Canal CW, Streck AF, Freitas LB, Delgado-Cañedo A, Bonatto SL, Salzano FM (2004). B-F DNA sequence variability in Brazilian (blue-egg Caipira) chickens. Anim Genet.

[CR30] Livant EJ, Ewald SJ (2004). High-resolution typing for chicken BF2 (MHC class I) alleles by automated sequencing. Anim Genet.

[CR31] Livant EJ, Brigati JR, Ewald SJ (2004). Diversity and locus specificity of chicken MHC B class I sequences. Anim Genet.

[CR32] Maniatis T, Fritsch EF, Sambrooke J (1982). Molecular cloning: a laboratory manual.

[CR33] McElroy JP, Dekkers JC, Fulton JE, O'Sullivan NP, Soller M, Lipkin E, Zhang W, Koehler KJ, Lamont SJ, Cheng HH (2005). Microsatellite markers associated with resistance to Marek's disease in commercial layer chickens. Poult Sci.

[CR34] Miller MM, Taylor RL (2016). Brief review of the chicken Major Histocompatibility Complex: the genes, their distribution on chromosome 16, and their contributions to disease resistance. Poult Sci.

[CR35] Miller MM, Bacon LD, Hala K, Hunt HD, Ewald SJ, Kaufman J, Zoorob R, Briles WE (2004). Nomenclature for the chicken major histocompatibility (B and Y) complex. Immunogenetics.

[CR36] Meziane EK, Potts ND, Viertlboeck BC, Lovlie H, Krupa AP, Burke TA, Watson KA, Richardson DS, Pizzari T, Göbel TW, Kaufman J (2019). Bi-functional chicken immunoglobulin-like receptors with a single extracellular domain (ChIR-AB1): potential framework genes among a relatively stable number of genes per haplotype. Frontiers Immunol.

[CR37] Moutsianas L, Gutierrez-Achury J (2018). Genetic association in the HLA region. Methods Mol Biol.

[CR38] Nguyen-Phuc H, Fulton JE, Berres ME (2016). Genetic variation of major histocompatibility complex (MHC) in wild Red Junglefowl (Gallus gallus). Poult Sci.

[CR39] Parker A, Kaufman J (2017). What chickens might tell us about the MHC class II system. Curr Opin Immunol.

[CR40] Phillips KP, Cable J, Mohammed RS, Herdegen-Radwan M, Raubic J, Przesmycka KJ, van Oosterhout C, Radwan J (2018). Immunogenetic novelty confers a selective advantage in host-pathogen coevolution. Proc Natl Acad Sci U S A.

[CR41] Potts ND (2016). Haplotype diversity and stability in the chicken major histocompatibility complex.

[CR42] Robinson J, Halliwell JA, Hayhurst JD, Flicek P, Parham P, Marsh SG (2015). The IPD and IMGT/HLA database: allele variant databases. Nucleic Acids Res.

[CR43] Rogers SL, Kaufman J (2008). High allelic polymorphism, moderate sequence diversity and diversifying selection for B-NK but not B-lec, the pair of lectin-like receptor genes in the chicken MHC. Immunogenetics.

[CR44] Salomonsen J, Marston D, Avila D, Bumstead N, Johansson B, Juul-Madsen H, Olesen GD, Riegert P, Skjødt K, Vainio O, Wiles MV, Kaufman J (2003). The properties of the single chicken MHC classical class II alpha chain (B-LA) gene indicate an ancient origin for the DR/E-like isotype of class II molecules. Immunogenetics.

[CR45] Scepanovic P, Alanio C, Hammer C, Hodel F, Bergstedt J, Patin E, Thorball CW, Chaturvedi N, Charbit B, Abel L, Quintana-Murci L, Duffy D, Albert ML, Fellay J, Milieu Intérieur Consortium (2018). Human genetic variants and age are the strongest predictors of humoral immune responses to common pathogens and vaccines. Genome Med.

[CR46] Shaw I, Powell TJ, Marston DA, Baker K, van Hateren A, Riegert P, Wiles MV, Milne S, Beck S, Kaufman J (2007). Different evolutionary histories of the two classical class I genes BF1 and BF2 illustrate drift and selection within the stable MHC haplotypes of chickens. J Immunol.

[CR47] Spurgin LG, Richardson DS (2010). How pathogens drive genetic diversity: MHC, mechanisms and misunderstandings. Proc Biol Sci.

[CR48] Trowsdale J, Knight JC (2014). Major histocompatibility complex genomics and human disease. Annu Rev Genomics Hum Genet.

[CR49] van Hateren A, Carter R, Bailey A, Kontouli N, Williams AP, Kaufman J, Elliott T (2013). A mechanistic basis for the co-evolution of chicken tapasin and major histocompatibility complex class I (MHC I) proteins. J Biol Chem.

[CR50] Van Kaer L (2002). Major histocompatibility complex class I-restricted antigen processing and presentation. Tissue Antigens.

[CR51] Walker BA, Hunt LG, Sowa AK, Skjødt K, Göbel TW, Lehner PJ, Kaufman J (2011). The dominantly expressed class I molecule of the chicken MHC is explained by coevolution with the polymorphic peptide transporter (TAP) genes. Proc Natl Acad Sci U S A.

[CR52] Walker BA, van Hateren A, Milne S, Beck S, Kaufman J (2005). Chicken TAP genes differ from their human orthologues in locus organisation, size, sequence features and polymorphism. Immunogenetics.

[CR53] Wallny HJ, Avila D, Hunt LG, Powell TJ, Riegert P, Salomonsen J, Skjødt K, Vainio O, Vilbois F, Wiles MV, Kaufman J (2006). Peptide motifs of the single dominantly expressed class I molecule explain the striking MHC-determined response to Rous sarcoma virus in chickens. Proc Natl Acad Sci U S A.

[CR54] Worley K, Gillingham M, Jensen P, Kennedy LJ, Pizzari T, Kaufman J, Richardson DL (2008). Single locus typing of MHC class I and class II B loci in a population of red junglefowl. Immunogenetics.

[CR55] Yaneva R, Schneeweiss C, Zacharias M, Springer S (2010). Peptide binding to MHC class I and II proteins: new avenues from new methods. Mol Immunol.

[CR56] Zoorob R, Bernot A, Renoir DM, Choukri F, Auffray C (1993). Chicken major histocompatibility complex class II B genes: analysis of interallelic and interlocus sequence variance. Eur J Immunol.

